# The chickpea genomic web resource: visualization and analysis of the desi-type *Cicer arietinum* nuclear genome for comparative exploration of legumes

**DOI:** 10.1186/s12870-014-0315-2

**Published:** 2014-12-18

**Authors:** Gopal Misra, Piyush Priya, Nitesh Bandhiwal, Neha Bareja, Mukesh Jain, Sabhyata Bhatia, Debasis Chattopadhyay, Akhilesh K Tyagi, Gitanjali Yadav

**Affiliations:** National Institute of Plant Genome Research (NIPGR), Aruna Asaf Ali Marg, New Delhi, 110067 India

**Keywords:** *Cicer arietinum* ICC4958, Clustering, Comparative genomics, Genome browser, Mapping

## Abstract

**Background:**

Availability of the draft nuclear genome sequences of small-seeded desi-type legume crop *Cicer arietinum* has provided an opportunity for investigating unique chickpea genomic features and evaluation of their biological significance. The increasing number of legume genome sequences also presents a challenge for developing reliable and information-driven bioinformatics applications suitable for comparative exploration of this important class of crop plants.

**Results:**

The Chickpea Genomic Web Resource (CGWR) is an implementation of a suite of web-based applications dedicated to chickpea genome visualization and comparative analysis, based on next generation sequencing and assembly of *Cicer arietinum* desi-type genotype ICC4958. CGWR has been designed and configured for mapping, scanning and browsing the significant chickpea genomic features in view of the important existing and potential roles played by the various legume genome projects in mutant mapping and cloning. It also enables comparative informatics of ICC4958 DNA sequence analysis with other wild and cultivated genotypes of chickpea, various other leguminous species as well as several non-leguminous model plants, to enable investigations into evolutionary processes that shape legume genomes.

**Conclusions:**

CGWR is an online database offering a comprehensive visual and functional genomic analysis of the chickpea genome, along with customized maps and gene-clustering options. It is also the only plant based web resource supporting display and analysis of nucleosome positioning patterns in the genome. The usefulness of CGWR has been demonstrated with discoveries of biological significance made using this server. The CGWR is compatible with all available operating systems and browsers, and is available freely under the open source license at http://www.nipgr.res.in/CGWR/home.php.

## Background

The draft genome sequence of the economically important pulse crop *Cicer arietinum* L. (chickpea; desi genotype) was recently completed via whole genome deep sequencing [1]. This initiative was undertaken by our group for the small-seeded chickpea genotype ICC4958 in view of the worldwide importance of legumes, drought-tolerant property of the genetic stock, and to facilitate genetic enhancement and breeding for development of improved chickpea varieties. The availability of the genome sequence of ICC4958 and the large-seeded kabuli-type chickpea [2] has led to enrichment of the existing volume of accessible legume sequence data that includes three other legume food crops, soybean (*Glycine Max*) [3], pigeonpea (*Cajanus cajan*) [4] and the common bean (*Phaseolus vulgaris*) [5], as well as two non-food legume plants, namely, the barrel medic (*Medicago truncatula*) [6] and birds foot trefoil (*Lotus japonicus*) [7]. Such a wealth of data enables a variety of comparative analyses and offers the legume research community an opportunity to develop tools for novel biological interpretations, paving the way for initiating new lines of research in legume genomics.

Despite a recent upsurge in chickpea genome research and despite the availability of draft sequences for two distinct chickpea genomes, there is a limitation of software available in the public domain for comparative exploration of these genomes, resulting in the absence of a comprehensive interface for genome analysis of any *Cicer* species, or for multi-genome data handling with respect to chickpea. To overcome this limitation, we have developed an interactive web server with feature-rich capabilities for detailed analysis and visualization of the chickpea nuclear genome, and it also supports detailed comparative genomics of ICC4958 with other chickpea genotypes (ICCV2, JG62 and PI489777, kabuli-type chickpea), legumes (pigeonpea, soybean, common bean, *Lotus* and *Medicago*) and non-legumes (*Arabidopsis* and grape). This web server has been named the ‘Chickpea Genomic Web Resource’ or CGWR and it is available freely without any login requirement at http://nipgr.res.in/CGWR/home.php. The CGWR includes a browser based on the Generic Genome Browser (GBrowse) [8] and multiple tools or interfaces for querying, analyzing, and downloading the available data. GBrowse is a web-server application implemented in Perl, highly suitable as a stand-alone genome browser, and is currently being used for more than 100 organismal genomes worldwide [8].

**Figure 1:**
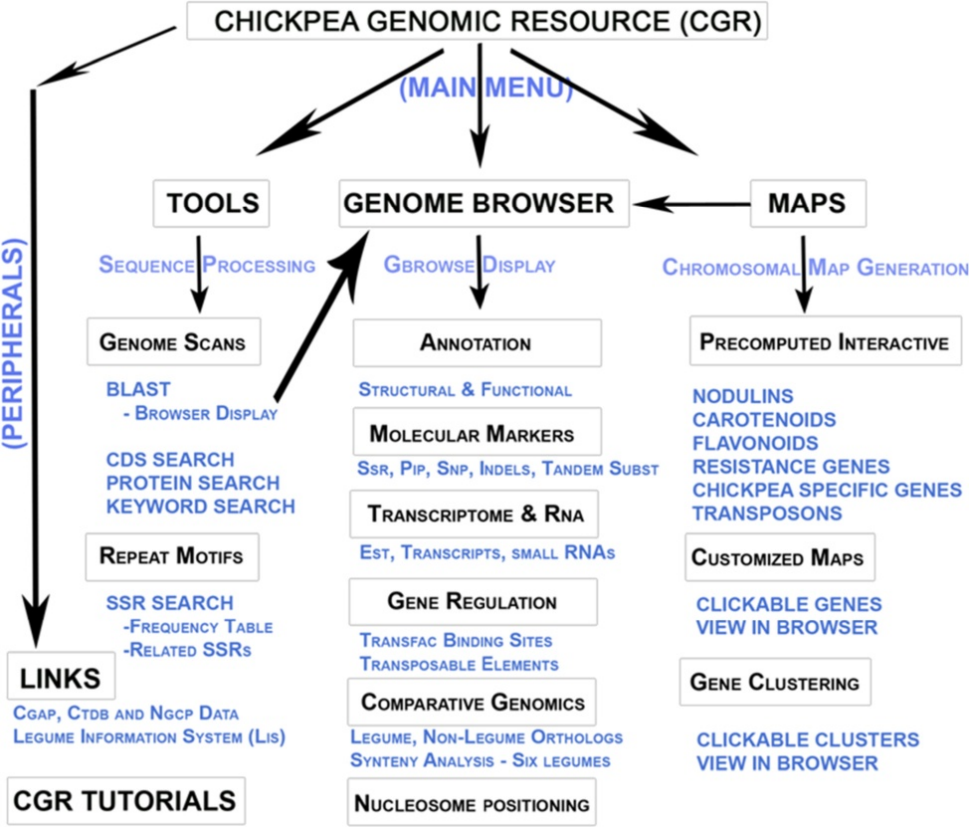
**A flowchart depicting overview of the CGWR components.**

This report provides a primer on the basic elements of the CGWR graphical user interface (GUI). For each menu, we provide a pipeline for routine tasks that can be performed using the CGWR with specific examples offering an insight into problems of biological interest that can be addressed through this resource, and finally we discuss our plans for the next CGWR release. Figure 1 provides an overall summary of the CGWR and its components. We believe that the CGWR will encourage researchers to perform legume-based informatics analyses as it is intended towards ease-of-use and interactive graphical display of many kinds of genomic information.

## Implementation

The CGWR has been split into three major sections. The first of these is the ‘Tools’ section, that enables rapid scanning of SSR repeats and extraction of desired CDS or gene as well as pair wise alignments, which aid in the identification of orthologous regions between species. This menu supports text and sequence based search providing quick and precise access to any desired gene or protein of chickpea. The second section is ‘Maps’, motivated by the need to have interesting single view snapshots of the chickpea genome map – the chromosomal location(s) of a desired gene model or gene family can be displayed by this tool in a clickable genes-based interactive image that is available for download as well. The ‘Browser’ within CGWR is a fast loading online tool for genome exploration and interpretation that provides a reliable display of a given region of interest in the chickpea genome at any scale, and enables data browsing, filtering and analysis through dozens of annotation ‘tracks’ in a single window. Apart from these three main sections, CGWR includes itemized tutorials for each section and provides important links to external legume research tools and websites, as well as the facility to download of all available datasets, thereby serving as a legume knowledge repository.

### Data sources

All data regarding the chickpea nuclear genome sequence, assembly, annotations, gene models and the reference sequence itself were generated under the NGCP project, as described in Jain et al. [1]. Genomic data for comparative analyses was taken from Phytozome v9.0 (http://www.phytozome.net/). Transcriptome data was obtained from NCBI and the CTDB database [9,10]. Gene ontologies were extracted from the *Arabidopsis* GO Database [11]. Nuclear genome assemblies for six other legumes were downloaded from their respective databases, viz. *Cicer arietinum* kabuli type genotype CDC Frontier (http://www.icrisat.org/gt-bt/ICGGC/GenomeManuscript.htm), *Cajanus cajan* (http://gigadb.org/dataset/100028), *Glycine max* (ftp://ftp.jgi-psf.org/pub/compgen/phytozome/v9.0/Gmax/assembly/), *Lotus japonicus* (ftp://ftp.kazusa.or.jp/pub/lotus/lotus_r2.5/pseudomolecule), *Medicago truncatula* (ftp://ftp.jgi-psf.org/pub/compgen/phytozome/v9.0/Mtruncatula/assembly/), and *Phaseolus vulgaris* (ftp://ftp.jgi-psf.org/pub/compgen/phytozome/v9.0/Pvulgaris/ assembly/).

### Comparative genomics and variations

For the Tools interface, command-line BLAST databases were created for *C.arietinum* ICC4958 draft Genome sequence, its peptides and CDS sequences. For the identification of orthologs and inter-species polymorphisms, BLAST databases were created for the eight plants listed above, as well as four varieties of chickpea. BLAST version 2.2.27+ [12] was used for this purpose. For every new run, the output gets converted to HTML and table format using PHP scripts. Backend perl scripting is used for integration of BLAST output with the genome browser to directly enable visualization of genomic region of the sequence of interest. The SSR search tool enables an overview of the number of iterations of any desired SSR motif of interest on the chickpea genome, through backend perl scripts. For identification of syntenic regions, a cut-off BLASTn score (<10^−10^) was applied between chickpea genome and the above six legume plant genomes. For computation of chains of syntenic regions, DAGchainer software [13] was used for which input files were prepared through inhouse processing scripts written in C++ and perl. Repetitive matches in the input files containing nine columns (chrA, accessionA, startA, endA, ChrB, acessionB, startB, endB and E-value) were removed in order to reduce data noise, and filtering was done taking 50 kb window lengths. DAG (Directed Acyclic Graph) and dynamic programming was used to compare each pair of genome sequences mentioned above.

### Mapping and clustering

Gene mapping and clustering data were generated using gene-location tables generated through PHP programming. The maps generated through this tool are interactive and use specific pseudomolecule based genomic locations of given gene IDs and arrange them in the order of occurrence on the eight LGs. An image is created with eight vertical bars, each representing one pseudomolecule (or linkage group), and gene models are marked on these bars as horizontal grey lines. Each horizontal mark in the map has been made clickable using shell and perl scripting, so that user can infer further details of the individual or group of mapped gene ids. For clustering, whenever two or more of the input IDs are found to lie within a pre-computed distance cut-off (0.3 Mbp) with respect to one another, the program assigns them to a cluster and returns a web link for the user to analyze this cluster further. Each gene model or cluster mapped to any of the eight assembled pseudomolecules can be directly visualized on the chickpea genome browser through a link that integrates the tools at the CGWR backend, as explained in the section above. In case, one or more input gene IDs do not map to any of the eight pseudomolecules or LGs, they are assigned to an ‘unassembled scaffold’ or a virtual LG termed as ‘UN’ which can be seen as the last (ninth) vertical bar on the map image. The program does not carry out clustering analysis of gene models mapped to this virtual pseudomolecule since the gene models are unassembled and their spatial locations are unknown.

### Regulatory element identification

Perl scripts were used for GFF file filtering, data normalization for removal of overlapping gene stretches, and for extraction of 300 bp upstream sequences for each annotated gene model. A total of 20,057 such sequences were obtained from the eight pseudomolecules and 18,826 scaffolds of *Cicer arietinum* nuclear genome and these were submitted to transcription factors binding site (TFBS) or *cis*-element prediction pipelines. Data on regulatory regions or *cis*-elements in the upstream regulatory regions of annotated chickpea gene models was obtained by computational prediction methods. For this, potential TFBSs were identified using PLACE [14] and JASPAR [15] databases, two programs that use distinct approaches, namely, literature-based, and position specific scoring matrix (PSSM) based methods, respectively. JASPAR contains annotated, matrix-based TFBS profiles for multicellular eukaryotes, derived from ChIP-seq and ChIP-chip whole-genome binding experiments. Briefly, the elements of a PSSM correspond to scores reflecting the likelihood of observing that particular position of the candidate TFBS. The parameters used for JASPAR CORE plantae included selection of eight plant species including 21 different transcription factors, each represented by a non-redundant profile, with an initial relative profile score threshold of 85%. The resulting data was refined using a score value of 7 to match the approximate lowest score obtained in the predicted TFBSs data file at 95% threshold. All the 102,597 hits obtained in this manner were incorporated into the CGWR browser using GFF3, PHP and Perl. PLACE is essentially a literature based database, containing curated and non-redundant nucleotide sequence motifs found in plant *cis*-acting regulatory DNA elements, extracted from previously published reports, articles, and reviews on the regulatory regions of various plants genes [14]. Mechanized perl modules were then used to obtain PLACE predictions by entering each of the 20,057 chickpea upstream regulatory region sequences into ‘PLACE Web Signal Scan’ grouped by signal.

### Nucleosome positioning maps

Predictions for nucleosome start sites and occupancy on the chickpea genome were carried out using a fortran based R package NuPop. The method uses a duration hidden Markov model with individual functions that compute the Viterbi prediction of nucleosome position; occupancy state and binding affinity score for a given stretch of DNA [16,17]. *Arabidopsis thaliana* was found to be the species with most similar base composition to chickpea, and thus nucleosome state predictions for chickpea were made using *Arabidopsis* model of pre-trained linker DNA length distribution. Among the parameters used was the 4th order Markov chain model for both nucleosome and linker DNA states. This model was found to be slightly more effective in prediction, although it required extra compute time (data not shown). Output of these predictions was converted to tab delimited files and thereafter to plots for visualization. For a genomics region to be considered in a likely nucleosome state, the criteria were delimited as follows: a minimum nucleosome start-site score (> = 0.45) followed by at least 146 base pairs, with high scores for nucleosome occupancy (Average > = 0.8). Regions that did not satisfy this criterion were treated as linker DNA states. In this manner, raw NuPop scores were converted to plots using in-house shell scripts for convenience of visualization. The track is presented as a plot, wherein regions with linker DNA states appear on the negative Y-axis while regions with high likelihood of nucleosome states appear on the positive Y-axis. Regions of the assembly that contain consecutive series of N’s are shown with zero score, to avoid confusion with predicted regions.

### Storage, extraction and GUI

All analyses were carried out as described, and results were converted to GFF format for storage, display and extraction. Back-end MySQL (version 5.5.29) was used to store all categories of data that enable sequence search and gene based mapping. GBrowse was used for construction and development of the genome browser, through the Generic Model Organism Database (GMOD) project, a collection of open source software tools for creating and managing genome-scale biological data [18]. For chickpea, GBrowse-2.27 was used along with Apache, standard perl libraries, libgd2 and MySQL on a Red Hat Linux platform and was configured to show both qualitative data such as the splicing structure of a gene, and quantitative data such as microarray expression levels. To improve responsiveness of the resource, the Apache configuration file was modified to replace the usual CGI implementation by the FastCGI protocol, and Perl FCGI modules were installed. For efficiency, features and sequences have been stored in a relational database whose modules and dependencies serve as the basis for data access in GBrowse. The data creation pipeline uses input data in two formats for browser operation, namely GFF and FASTA, both inter-convertible through bioperl modules. The backend data loading pipelines use MySQL and a tab-delimited file containing the various genomic features in GFF format along with bioperl tools for loading Bio::DB::GFF databases. Overall CGWR configuration and customization has been performed through FCGI, Javascript, PHP and HTML scripting. Front-end pages were generated using HTML scripts. Different in-house PHP and perl programs were written to create the output. Mapped images are generated using CPAN modules GD, ChromosomeMap-0.10 and ImageMagick-6.8.5-6 (http://www.cpan.org/). In order to make the mapping module of the CGWR more robust, shell scripts have been added which can allow multiple users to access and visualize mapping results simultaneously.

## Results

This work is focused on the comparative genomic analysis of the draft nuclear genome assembly of *Cicer arietinum* genotype ICC4958 as published by our group recently [1]. At the top level, the current assembly is organized as Ca_LG_1 to Ca_LG_8; representing WGS contigs matched to the eight chickpea linkage groups, while Ca_LG_0 represents scaffolds that could not be matched to any of the eight pseudo-molecules. The chickpea genomic resource can be accessed through the webpage http://nipgr.res.in/CGWR/home.php. Figure 1 provides a flowchart summary of the CGWR and its components. In the following sections, we describe the main menu items individually followed by a brief account of the available tools and genomic tracks in the browser (represented as colored, collinear blocks with text labels and strand annotation) along with their salient features.

### CGWR tools

**Figure 2:**
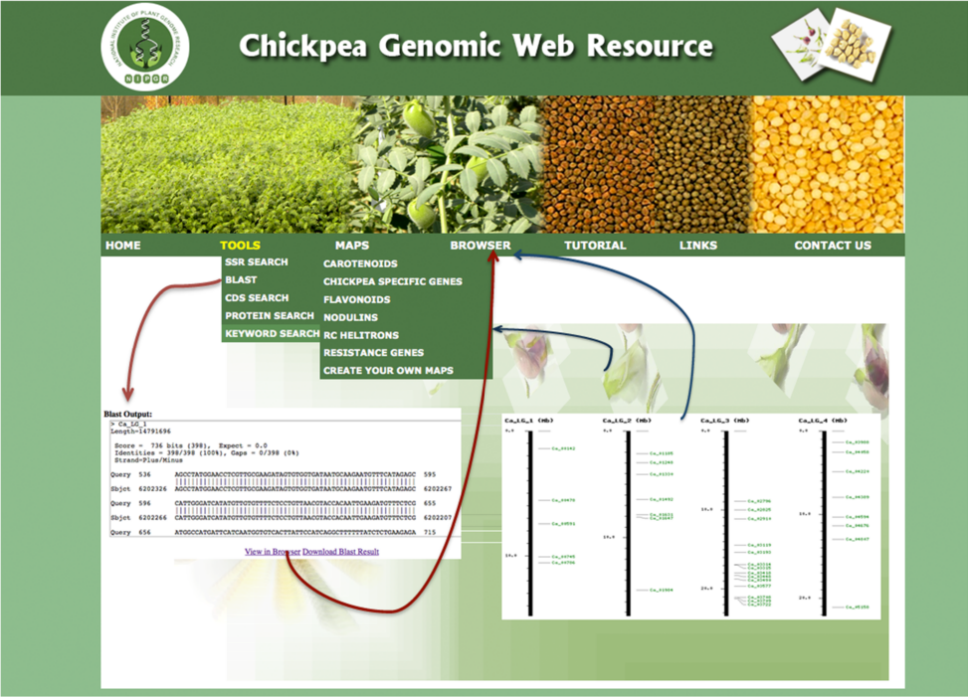
**The CGWR home page containing an outline of its features and capabilities.** Insets depict typical outcomes of BLAST and Mapping and clustering runs showing links to genome browser for visualization of the gene or cluster of interest on the chickpea genome.

The Tools menu of CGWR comprises a simple user-friendly GUI that enables rapid scanning and extraction of desired regions of the genome as well as pairwise alignments with user specified sequences, for identification of paralogs and orthologs. Various options are available to users from this pull down menu, including SSR Search, BLAST, CDS, Protein and Keyword Search, as shown in Figure 2.

### SSR search

This page enables microsatellite analysis for 64418 simple sequence repeats (SSRs) detected in chickpea genome through *in*-*silico* identification. It contains a form where users can provide the motif of an SSR of interest, such as ACA. The tool finds all SSRs in the chickpea genome that match an input string and split the data for ease of interpretation resulting in a table with frequency of occurrence at individual iterations of the SSR. For example, ACA repeats occur at a total of 231 locations in chickpea nuclear genome, of which five iterations of ACA (i.e. ACAACAACAACAACA) occur at 112 positions whereas ten iterations (ACA^10^) occur at only three positions, and so on. At the bottom of the results page, this tool also returns an assessment of all ‘related’ SSR sequences that are one or two nucleotides longer than the input SSR sequence. For the ACA example, ‘related’ SSRs would include all instances of ACAX, XACA and XACAX, data for which gets scanned and reported along with the repeat number and frequency. (X here, refers to any of the four nucleotides A/C/T/G).

### BLAST

The Basic Local Alignment Search Tool is a commonly used alignment program for detecting sequence similarity. Users can select the specific BLAST program and database based on the nature of their query which may be DNA, protein, translated RNA, or translated DNA. Database options for this tool include the complete set of CDS and proteins for ICC4958 as well as the nuclear genomic sequence. Input sequence can be entered as text, in FASTA format, or multiple sequences can be uploaded as files, if required. The tool returns alignments in HTML format and a summary of the output can be downloaded. In addition to text based summary, this page directly connects the tools menu to the chickpea browser within CGWR through a ‘browser’ link, as shown in Figure 2, to enable detailed investigation of the genomic region of interest. Upon every BLAST search, an additional ‘Alignment Track’ labeled “BLAST” gets added in the Browser for users to see the exact region of the genome aligned to the query, without any requirement for manual intervention.

### CDS search

Apart from BLAST, the CGWR also enables direct Coding DNA Sequence (CDS) search for a known gene model, ID of which can also be identified by a BLAST search, as described above. The coding region of a gene is the portion composed of exons, and codes for protein. For an organism, it represents the sum total of the genome that is composed of gene coding regions. All CDSs predicted computationally for chickpea [1] can be searched by this tool, where users can paste one or more IDs of interest and obtain the respective CDSs. Results are directly connected to the chickpea browser to enable detailed investigation of the genomic region of interest, as explained above.

### Protein search

Similar to the CDS search, the computationally predicted complement of translated regions for the chickpea genome (as per ref [1]) can be scanned by ID number. This menu supports text-based search providing quick and precise access to any desired protein of chickpea. Results can be downloaded and directly visualized in the genome browser, with examples provided within the form.

### Keyword search

In case users do not have any prior information such as the sequence of interest or CIDs, the keyword tool allows a search of all potential chickpea IDs that contain a given input string of text in their annotation. For example, the tool returns six potential matches to the term ‘reductoisomerase’, and the list of these six can be downloaded along with information of each matched ID, including gene description, locus, PFAM ID and GO slim term. Further, the CGWR algorithm automatically generates an interactive map for the searched query, so that users can directly visualize the spatial patterns of occurrence of the list of IDs obtained from their search.

### CGWR maps

The Maps menu provides interactive chromosomal maps of gene families, i.e. locations of desired gene models on their respective pseudomolecules. This tool produces genomic, sequence-based maps and displays pseudomolecules with the coordinates being in base pairs. It can also be used to click on any desired gene or cluster on the map in order to evaluate and visualize clustering of the mapped gene models on the chickpea genome. Users can obtain interesting single view snapshots of the chickpea genome wherein spatial position(s) of requested gene(s) can be displayed simultaneously across the eight pseudomolecules. This menu offers two procedures, one for visualization of pre-existing maps for selected chickpea gene families, and the other for customized construction of maps for desired sets of gene models by the user.

### Chickpea gene family maps

**Figure 3:**
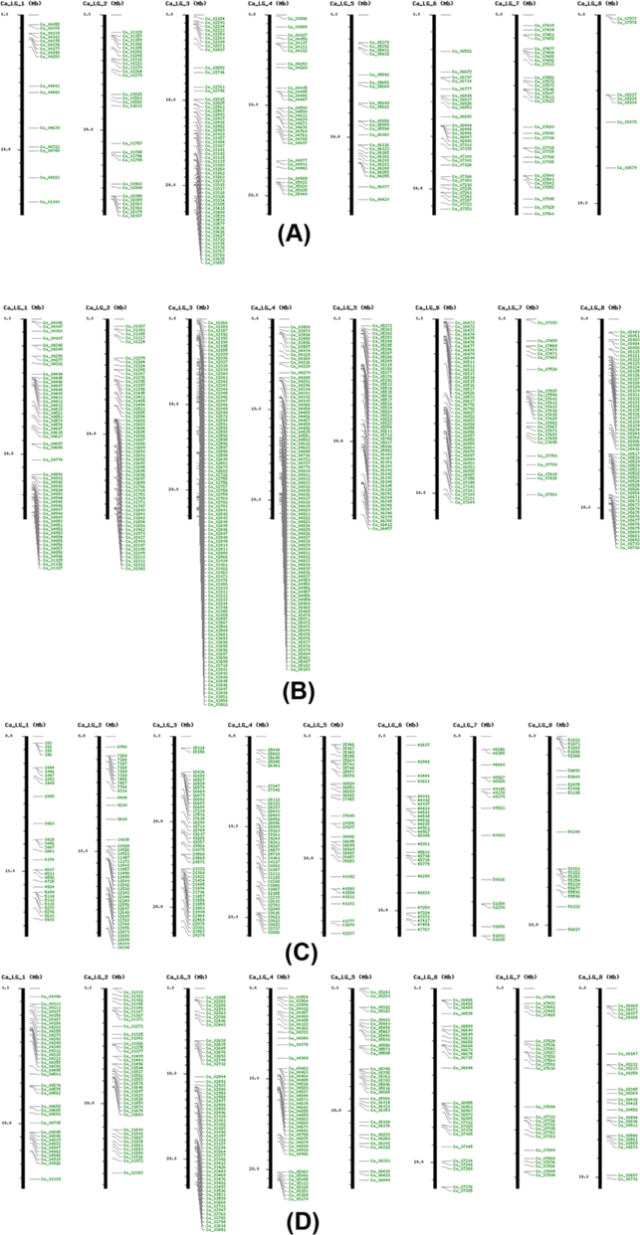
**Genome maps of various chickpea gene families. (A)** Flavonoids **(B)** Chickpea-specific gene models **(C)** DNA Transposons - RC Helitrons **(D)** R-genes. In each panel, vertical bars represent the eight distinct chickpea pseudomolecules (LGs), while individual members of respective gene families are marked in red horizontal lines on each bar, corresponding to genomic locations.

Of the 640 unique gene models identified to be associated with the metabolism of flavonoids in chickpea, those that could be mapped to the eight distinct pseudomolecules have been depicted in Figure 3A, and the highest number of flavonoid gene models were found clustered on pseudomolecule 3. Such a tendency to cluster was not observed for gene models predicted to be associated with carotenoid metabolism (data available on CGWR website under Maps menu). Each gene or cluster can be analyzed in detail by clicking on the respective bar on the map image. For example, the top three flavonoid genes on LG-3 fall into one cluster that can be clicked to see full details of each member of the cluster, including gene name, functional annotation, gene ontology, TF binding sites, and complete sequence. More information can be noted by clicking the link that connects each gene or cluster to the CGWR genome browser. Our analysis across the entire plant kingdom revealed 9990 legume specific gene models and 2751 chickpea specific gene models in the chickpea genome and panel B of Figure 3 shows the mapped subset of the chickpea specific genes. Further, the putative resistance related gene models (R-genes) as identified through screening of the chickpea unigene set were also mapped and these appear to reside throughout the chickpea genome, although clustering may occur within the specific conserved classes that R-genes were assigned during the analysis (Figure 3D). Almost one third of chickpea genome repeats were identified to be various kinds of transposable elements, a majority of which represented retrotransposons and about 5% constituted DNA transposons. Of the latter group, Figure 3C shows the mapped RC helitrons, i.e. transposons that are thought to replicate by a rolling circle mechanism, and it can be seen that they are interspersed all over the chickpea genome and clustered in a few regions. It is notable that several types of LINEs and other gene families also appear to be clustered on the chickpea genome and it may be interesting to find out whether the clustering occurs in other legume genomes as well. This possibility can be queried within the CGWR by using a combination of the browser and tools menu as described in the following sections.

### Customized maps

**Figure 4:**
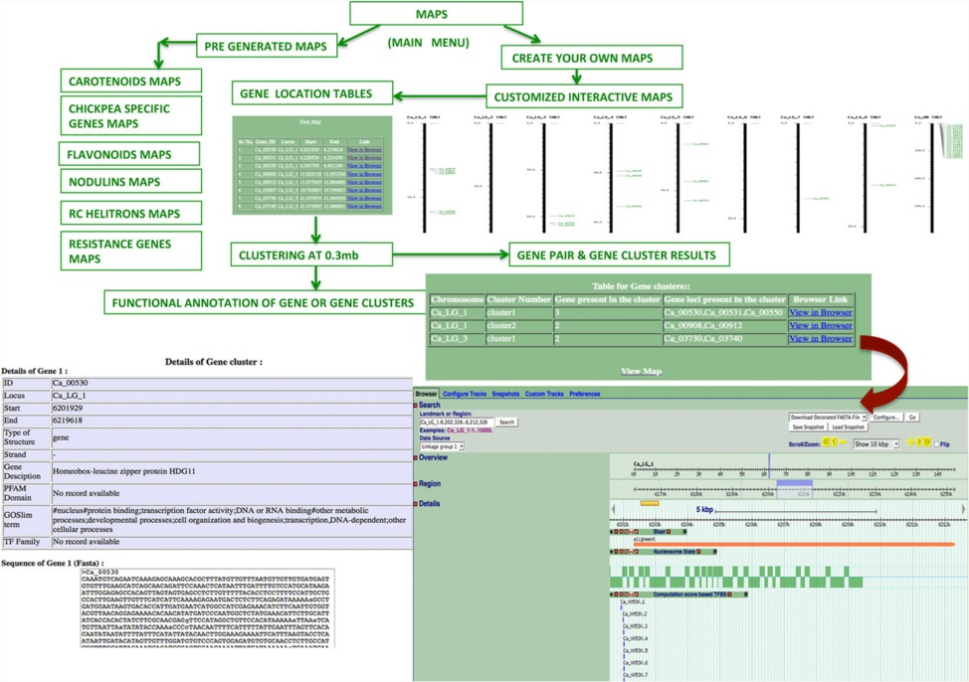
**The Maps Tool of CGWR.** This tool can be used for generating customized genome wide interactive maps of genes and gene families of interest. Six kinds of pre-generated maps are available, along with clustering options. Seamless integration with the chickpea genome browser as shown in the lower right panel enables further analysis.

Figure 4 shows a flowchart based outline of the Maps Tool in the CGWR. The ‘create your own map’ option allows users to paste the IDs of their desired set of gene models to visualize spatial location maps similar to the ones depicted in Figure 3. On the submission form, users can type the gene ID into the input box, and hit enter on the keyboard. An example set of gene IDs is provided within the form itself. Users can also determine the CIDs of genes of interest through the keyword search. As shown in Figure 4, the map tool returns a table listing out the loci, start and end positions of the specified gene IDs provided as input. At the top of this table is a link to view Map that leads to the mapped image. If the gene of interest lies on one of the unassembled scaffolds, rather than one of the eight pseudomolecules, the program assigns it to an independent unassembled unit or virtual LG termed as ‘UN’. An example of such a case is provided within the CGWR pre-generated maps. These custom generated maps are interactive, allowing users to click any region of interest on the map, to visualize details about the respective region as described in the previous section. In addition, users can directly find links to the chickpea browser from any of the input gene models mapped by this algorithm, as clicking on these links redirects users to the corresponding regions of the chickpea genome, as shown in Figure 4. These maps can also be downloaded as high-resolution images for publication purposes. Thus, the CGWR provides direct connection between its various features by connecting Tools, Maps and the Browser at its backend.

### Gene clustering

As shown in Figure 4, whenever two or more mapped gene models are found to occur within a pre-computed distance cut-off with reference to each other, they are considered to be part of a physical genomic cluster. In such cases, the output of the map will provide an additional ‘Clustering’ link. With the help of this feature, users can directly visualize the number of clusters and composition of each cluster identified in the input set of gene models. The maps are interactive and each cluster on the map can be clicked manually for gaining insights into its members, while users can also view the entire genomic region containing such gene clusters on the chickpea genome browser, for further analyses as shown in Figure 4, such as the presence of common upstream regulatory elements, or to identify nearby gene models and their functions.

### Chickpea genome browser

**Figure 5:**
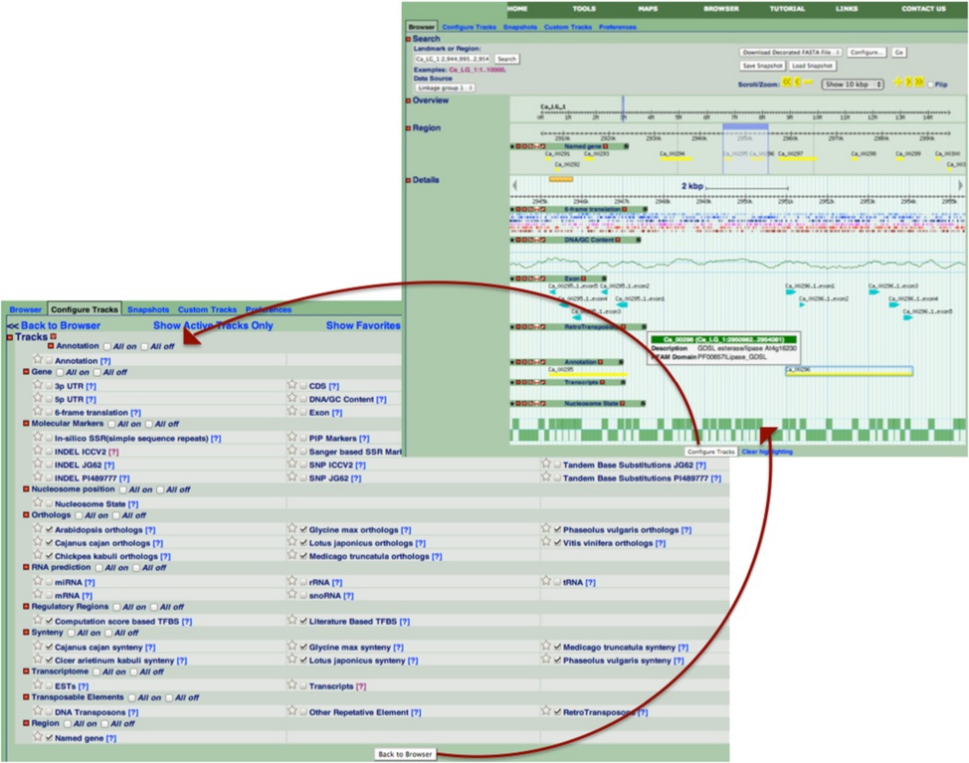
**Typical display of the chickpea genome browser in the region of the first LG.** Four main areas can be seen on the top left side of the upper panel panel, namely, Search, Overview, Region and Details. The topmost ‘Search’ section identifies the exact genomic range displayed in the browser (see ‘Landmark’ textbox on top left corner). The area highlighted in sky-blue shades in both of next two sections, namely, ‘Overview’ and ‘Region’, is expanded in the remaining browser view. Accordingly, the current example (‘Details’ section) represents a 10 kbp stretch within 3 Mbp region of CA LG 1. The 3 Mbp region has about 11 gene models (see yellow bands in ‘Region’ Section), of which only two lie within the expanded Details section (see yellow bands in Annotation track). Annotation of the gene models can be seen by clicking the annotation bands in the expanded section, in the form of a pop-up box, as shown here. In this image, seven genomic tracks have been toggled on, including retrotransposons, nucleosome states, and the transcriptome. Users can select additional tracks from over forty-eight options in the present CGWR build, as shown in the lower left panel.

The genome browser is one of the primary capabilities of the CGWR. Currently, the May 2013 assembly is available; the next freeze of the assembly will be made accessible as soon as it is released, in the near future. Figure 5 shows the default browser display i.e. the first 10 kbp data on the first Chickpea pseudomolecule LG1, although users can select any of the eight LGs from the pull-down list in the Data Source, and positional information can also be typed into the landmark or position box on the top left corner, e.g., Ca_LG_1 for the whole of chromosome 1 and Ca_LG_2:1..10,000 for the region from position 1 to 10,000 on chromosome 2. The region expanded in the browser will be highlighted in pale blue in the Overview section as shown in Figure 5. For the unassembled scaffolds, users can select Ca_LG_0 from the pull down data source list in the Search section, and type the name and position of the desired scaffold. Zooming and scrolling controls help to narrow or broaden the displayed chromosomal range to focus on the exact region of interest. Default browser display can be altered as desired by using track controls offered at the bottom of the browser enabled through the ‘configure tracks’ button, where about fifty different tracks are available to choose from, as shown in Figure 5.

In order to avoid information overload on account of such a large number of tracks, GBrowse controls can be coordinated in such a manner that display for some browser tracks may be turned off, and others may be collapsed into a condensed single-line display. Tracks can thus be hidden or filtered according to user preferences using track-based toggles for on/off and hide/show modes, apart from download, share, density and favorite modes. There is also a configure mode on each track that allows users to edit the display characteristics with respect to that track. Hovering on the colored bar corresponding to each track display releases an information bubble describing the respective track, and its data source(s), wherever applicable. Clicking on individual colored bars or features within a track opens a details page containing a summary of the respective properties of the track, with additional feature-specific information such as alignments or links to external information depending on the nature of the track. In the following section, we provide a list of tracks and examples of typical cross-track analyses that the CGWR browser can be used for.

### Gene structure prediction

Currently the browser has seven independent tracks for genes and gene predictions that describe various aspects of gene structure, including tracks for selecting 5′ and 3’ UTRs, coding region (CDS), exons and introns for genomic DNA. The mRNA sequence for the predicted protein sequence is also available, along with GC content and six-frame translations of the genomic DNA.

### Functional annotations

For protein or RNA coding genes, functional annotations are provided in the ‘Region’ and ‘Details’ sections of the main browser window. The uppermost ‘Named Gene’ track within Region section allows visualization of gene models outside the user-selected highlighted area expanded in all subsequent (lower) tracks. For visualization of gene annotation within the user-selected highlighted region, the ‘Annotation’ track can be used. These gene models are in yellow bars, and mouse hovering will open a bubble with functional annotation and PFAM domain information, wherever available. Clicking on each gene will return a page with detailed locus information, gene description, protein family classification and gene ontology Information, as well as the nucleotide sequence of the respective gene in FASTA format.

### Molecular markers

The CGWR has a total of 12 individual tracks for assessment of molecular markers at the genomic level in chickpea. These include simple sequence repeats of two kinds, namely, *in*-*silico* SSRs and sequencing based SSRs, PIP markers, as well as tandem base substitutions and indels with reference to three other chickpea varieties. A total of 1,644,016 markers are depicted in these tracks. All SSRs identified on the genome can be visualized through an SSR track that enables further data analysis of various kinds. Hovering over an SSR will specify the number and type of that repeat, as to the number of SSRs of that specific kind present in the genome. For example a given SSR may be the fiftieth tetrameric SSR or the thousandth dimeric SSR etc. Clicking on the SSR will return a page detailing locus information, type, length, number and iteration of the SSR, along with the exact SSR motif. This track also has the facility to obtain the DNA from the flanking regions of the feature including 100 up- and down-stream bases to enable primer design efforts. In addition, the CGWR browser enables further interactive SSR analysis wherein users can find the number and type of any desired SSR. This page contains a form where length and motif of the SSR of interest can be typed in, and it returns a table providing information about whether SSRs of the respective kind are present, and if so, the number of SSRs in the concerned chromosome will be depicted as well. The SSR search options in the pull-down ‘Tools’ menu on the home bar at the top of the browser further enables a scan of all kinds of ‘related’ SSRs that differ by a length of one or two form the input SSR, as described earlier. Nucleotide diversity has been measured at the genomic scale by comparing ICC4958 with three other cultivated and wild chickpea genotypes, namely, desi-type JG62/ICC4951, kabuli-type ICCV2/IC12968, and wild-type P1489777. Variations have been analyzed between these four varieties revealing 32,919 InDels, and 1,504,646 SNPs and 41,824 tandem base substitutions all of which can be browsed in the CGWR through nine individual tracks representing each of these three categories compared pairwise between IC4958 and one of the above-mentioned genotypes. Each track, when clicked, provides details of gene structural variation via alignments between IC4958 and the respective variety being compared, along with additional flanking alignments from upstream and downstream regions, in order to assist in marker based studies and for acquiring the DNA for additional features and reverse complementation. The potential intron length polymorphism marker track (PIP markers) shows the markers that have been predicted using the PIP database.

### Comparative genomics

As highlighted earlier, the most important and voluminous data in CGWR represents the comparative assessment of chickpea genome with other leguminous as well as non-leguminous plant species. In all the CGWR comprises 24 individual tracks for comparative genomics, of which, nine tracks representing nucleotide variation between four chickpea genotypes have been described above under molecular marker section. Additionally, there are nine more conservation tracks for depiction of orthologous gene models between ICC4958 and six other legumes including the kabuli genotype chickpea ‘CDC Frontier’, *Glycine max*, *Medicago truncatula*, *Phaseolus vulgaris*, *Cajanus cajan*, and *Lotus japonicus*, apart from *Arabidopsis thaliana* and *Vitis vinifera* (both non-legumes). These ortholog tracks show measures of evolutionary conservation and highlight regions of the genome that may be functionally important between the pair being considered. Clicking on the track leads to details of locus position, gene IDs and FASTA format sequences for both orthologous gene as well as the chickpea gene under consideration. At the bottom of this page is a link to the alignment between the orthologs. The BLAST [12] search engine described earlier in the Tools menu is also available to meet specific needs of comparison and alignments. Apart from these 18 tracks, CGWR also has six tracks for synteny evaluation of genotype ICC4958 with each of the six legume genomes listed above. We recommend a large window size to enable visualization of the direction of synteny for a given region, as well as to explore multiple syntenic matches between two genomes in a given region. Clicking on the synteny regions pops up a bubble that provides the start and end site for each matched locus. Color codes have been maintained for each plant species across the 24 comparative genomic tracks. Blue, for example, represents *Phaseolus vulgaris* while red represents *Cajanus cajan*, and so on.

### Transcriptome

The browser contains tracks for detailed transcriptome analysis, with over 27000 ESTs and 274 million filtered reads representing transcripts of chickpea from independent tissue/organ based samples. The track for EST returns locus and strand information along with full nucleotide sequence of the EST. For each transcript, the track provides locus information, transcript description, data from gene ontologies of molecular function, biological process and cellular location, apart from enabling users to view expression across each of the six tissue samples studied.

### Regulatory regions

Specific binding of transcription factors (TFs) to short and degenerate oligonucleotides on the genome is key to transcriptional regulation and gene expression. The CGWR browser contains tracks for predicted TF binding sites (TFBS) according to both PSSM based computational scores (JASPAR track) [15], as well as literature based data correspondence (PLACE tracks) [14]. For each track, the browser provides information regarding strand, locus, TFBS sequence motif, family-based classification as well as the plant species with evidence of a similar binding site. The family based classification allows one to decipher what transcription factor might bind to the region of interest and CGWR further provides details of the identifier and each family. For example, the site ‘CAACTC’ is known to bind to transcription factor CAREOSREP1, and is from the family of CAREs (CAACTC regulatory elements) found in the promoter region of a cysteine proteinase (REP-1) gene in rice. Regulatory region analyses can usually result in multiple TFBS predictions for the same site and therefore incorporation of two independent tracks for this purpose in CGWR provides the additional advantage of cross-referencing and evidence from multiple sources. We recommend a low window size in the range of 5 to 10 kb in order to visualize multiple predictions in an individual manner.

### Transposable elements

Over 40% of the assembled draft genome represents interspersed repeats including various classes of transposable elements and these can be displayed individually. These tracks are available for DNA transposons, retrotransposons (LTRs) and other repetitive elements. The track for DNA transposons can be used to visualize more than 80 different kinds of DNA transposons as well as RC Helitrons. The track for retrotransposons enables visualization and analysis of various families of LINEs, SINEs and LTRs. Elements that could not be be classified into either of these two tracks have been assigned to a third track within transposable elements, namely, the other repetitive elements track, which consists of unknowns, simple-repeats, satellites etc. Each transposable element has been assigned a unique ID based on its genomic position. Transposable element IDs that occur in multiple copies with identical scores have been assigned sub-ids such as N.1, N.2, N.3 and so on, depending upon the number of occurrences. Clicking on an element will return a page detailing locus information, type and family-based classification of that specific transposon or retrotransposon, along with its entire sequence.

### Non-coding RNA predictions

Gene models for non coding RNAs have been predicted for the chickpea genome, resulting in identification of about 121 distinct Rfam families including miRNA, snoRNA, rRNA, tRNA etc. These have been mapped to the genome and can be visualized via five individual tracks. Clicking these tracks returns a page containing features of the respective RNA locus, such as anticodon and amino acid specification (for tRNA), unique family classification (for miRNAs and snoRNAs), strand information; complete sequence as well as 2-dimensional structure notation.

### Nucleosome positioning

Predictions for nucleosome states and linker DNA states for chickpea have been made using *Arabidopsis* as index species [16,17], and results have been normalized and mapped to the chickpea genome as described in methods. This track provides a plot with information about nucleosome and linker DNA states. It superimposes occupancy and binding affinity scores, Viterbi predictions for optimal nucleosome positioning and the posterior probability of a genomic position to be the start of a nucleosome. For convenience of interpretation, regions of the genome that have a higher tendency toward nucleosome states are depicted on the positive Y-axis, while regions with higher tendency towards linker DNA states are shown on the negative Y-axis. A preliminary computational correspondence between nucleosome occupancy likelihood and gene structure reveals that coding regions of the genome are significantly enriched for nucleosome states than the regulatory regions, while the promoters are significantly depleted of nucleosomes. We also find that introns have much higher density for nucleosome states than any other genomic region (data not shown). These and other interesting aspects of the chickpea nucleosome positioning predictions are currently being investigated further in our laboratory.

### Component integration and other services

In order to facilitate seamless exchange of data between its various components and capabilities, the CGWR backend enables dynamic inter-connections and frequent coupling of results between its three main sections, the Tools, Maps and the genome Browser. For example, users can carry out a BLAST run for identification of orthologs of their sequence of interest, and use links on the output page for direct access to the genomic regions containing the orthologs of interest. The list of potential orthologs can also be obtained by typing in a keyword of interest. The IDs thus identified can be mapped to the assembled genome for a high-resolution interactive image via the Maps menu, where again, direct connection to the browser is provided. For instance, the gene IDs of the chickpea orthologs obtained in the BLAST search (under Tools menu) can be pasted into the Maps menu to visualize where these domains lie on the chickpea genome, and whether they show any tendency towards spatial clustering. Whenever a gene of interest is found to lie within close proximity of other gene models of the same family, it is assigned to a cluster that can be visualized on the interactive map as well as the CGWR browser for detailed analysis of other aspects of the clustered region. Apart from these backend provisions, the Links menu of CGWR provides access to a wide variety of datasets and links to important external information regarding chickpea and legume-based genomics research. This menu also enables downloads of various datasets used in this work.

### Example of a typical analysis

A user may have a gene of interest for which they want to find legume and non-legume homologs. It is possible to start by using BLAST of the sequence of interest against the chickpea genome in the Tools menu, and find the link to the browser on the BLAST output page. This will lead to the chickpea homologous gene in the browser display, and from here, similarity with six legumes and two non–legume plants can be found by using one of the 15 pre-computed comparative genomics tracks. Alternatively, nine pre-computed tracks enable detailed nucleotide level assessment of structural variations between chickpea and its wild- and desi-type cultivars. Clicking on these tracks will enable viewing comparative alignments as well as information about the co-ordinates of the alignment on both genomes. In case multiple homologs of the gene of interest are found within chickpea, these can all be mapped using the Maps menu for interactive and convenient detection of clusters within the respective gene family. The clusters, if any, can be further analyzed for other features of interest in and around the region via integrative links between maps and the genome browser. For example, users can toggle the ortholog tracks for the cluster to find out whether the concerned set of gene models is clustered in any of nine other plant genomes as well. Shared transcription factor binding sites, if any, in the upstream regulatory regions of clustered gene models can also be visualized through designated tracks in the browser. With the EST, mRNA and transcript tracks visible, it is possible to see the extent, if any, of tissue specific or organ specific expression for these gene models in chickpea. Additionally, turning on some of the TFBS prediction tracks would suggest whether there is evidence for any specific TF binding to the promoters of the identified homologs. Patterns of nucleosome arrays can also be visualized for assessment of DNA accessibility in these regions of the genome, and compared for overlap with other features such as coding and non-coding areas.

## Discussion

The construction and development of the Chickpea Genomic Web Resource was undertaken to enable the legume community to carry out coherent informatics-based exploration of the recently sequenced chickpea nuclear genome, including mapping and visualization of structural and functional aspects of the underlying DNA sequence. The CGWR offers a wide-ranging analysis of the chickpea genome, along with options to carry out comparative cross-species studies of eleven other species or genotypes with respect to chickpea, and is the only plant-based resource offering customized and interactive chromosomal location maps for desired gene-models and gene-families. Furthermore, the browser within CGWR is the only plant genome browser that offers display and analysis of nucleosome positioning patterns, revealing instances of regularly spaced arrays of nucleosomes along the coding regions, along with a tendency for the upstream regulatory regions to be relatively depleted of nucleosomes. CGWR results can thus provide opportunities for future research endeavors on comparative analysis of nucleosome maps and how their genomic arrangement may control gene regulation. Another unique feature of the CGWR is exhaustive information on *cis*-regulatory element data in the upstream regions of the chickpea nuclear genome, based on distinct prediction approaches. Most of the existing plant genome browsers provide conventional data on gene models, transcripts, duplication, syntenic regions and repetitive elements. In numerous cases, even this information is unavailable. For example, a chickpea genome browser is presently hosted by the Legume Information System (LIS) at http://cicar.comparative-legumes.org/gb2/gbrowse/Ca1.0/ [19]. It provides access to genome (version 1.0) and transcriptome assemblies (version v2) for the CDC-Frontier (kabuli-type) chickpea genotype, but there is no support for intra- or inter- genome analyses. Another database specific to cool season food legume genomes is available at http://coolseasonfoodlegume.org/ [20]. CSFL is highly informative in terms of legume based QTLs, and includes browsers and marker search tools for pea, lentil, chickpea and *Medicago*, although it has no data on desi-type chickpea, and its kabuli-type genome browser has less than ten tracks with no possibility for comparative genomics with any of the other cool season food legumes. However, this comparative technical discussion of both LIS and CFSL does in no way undermine either resource, as they have a large number of advantages, including state-of-the-art visualization techniques, highly advanced QTL search options and regular global updates on legume related news and workshops, providing substantial benefits for the community. In summary, we believe that CGWR fills a different niche, rather than create redundancy by competing with other platforms, and therefore it would be welcome by the legume community. The innovative approach implemented in the CGWR would pave the way for in-depth display and interpretation techniques for diverse kinds of biologically relevant data, thereby inspiring multiple future research initiatives. Complete documentation and online tutorials are also available within the CGWR, for understanding the resource and its capabilities.

## Conclusions

The CGWR is a multi-faceted web resource for dynamic analysis and specialized visual inspection of the most recent draft assembly of the chickpea nuclear genome. It is freely available without any login requirement at http://www.nipgr.res.in/CGWR/home.php. Each new draft assembly of chickpea will be integrated into the CGWR and made available as it is released. In future versions, we hope to modify and expand the browser to add new features of interest to the legume research community, such as incorporation of biochemical pathways, simultaneous visualization of multiple gene family maps in distinct colors and tracks for comparative genomics with many more plant species. Further, we hope to integrate the organellar genomes into the CGWR to enable a more complete global analysis. We welcome and encourage suggestions for new and interesting tracks from our users.

## Availability and requirements

**Project name:***Chickpea* Genomic Web Resource (CGWR)

**Project home page:**http://www.nipgr.res.in/CGWR/home.php.

**Operating system (s):** Platform independent

**Programming language:** Perl, PHP, FORTRAN, CGI

**Other requirements:** Nil

*License*: Open Source license GNU GPL v2

*Any restrictions to use by non*-*academics*: Available Freely to The Community

## Abbreviations

BLAST: Basic local alignment sequence tool
CA_LG: *Cicer arietinum* linkage group
CDS: Coding DNA Sequence
CGI: Common Gateway Interface
CGWR: Chickpea Genomic Web Resource
Contig: A continuous sequence of DNA that has been assembled from overlapping cloned DNA fragments
CPAN: Comprehensive Perl Archive Network
CTDB: Chickpea Transcriptome Database
DNA: Deoxyribonucleic acid
EST: Expressed sequence tags
GFF: Generic Feature Format
GFF3: Generic Feature Format Version 3
GMOD: Generic Model Organism Database
GUI: Graphical user interface
GBrowse: Generic Genome Browser
HTML: HyperText Markup Language
LINEs: Long Interspersed Elements
LTRs: Long Terminal Repeats
miRNAs: MicroRNAs
MySQL: Structured Query Language
NCBI: National Center for Biotechnology Information
NGCP: Next Generation Challenge Programme
PFam: Database having large collection of protein families
PHP: Hypertext Preprocessor
PSL: Text file format for representing sequence alignments
PSSM: Position-Specific Scoring Matrix
RC Helitrons: Rolling circle Helitrons
RNA: Ribonucleic acid
rRNAs: Ribosomal RNAs
SINEs: Short Interspersed Elements
snoRNAs: Small nucleolar RNAs
SNP: Single nucleotide polymorphisms
SSR: Simple Sequence Repeat
STS: Sequence Tagged Site marker represents a single, unique, sequence-defined point in a genome
Supercontig: A supercontig consists of one or more sequence contigs known to occur in a specific order and orientation
TFBS: Transcription Factor Binding site
tRNAs: Transfer RNAs
WGS: Whole genome shotgun sequencing method
